# Supplementary material for: Dynamic Evolution of Humoral and T-Cell Specific Immune Response to COVID-19 mRNA Vaccine in Patients with Multiple Sclerosis Followed until the Booster Dose
Source: Int J Mol Sci. 2023 May 10;24(10):8525. doi: 10.3390/ijms24108525 (PMC10218688; doi:10.3390/ijms24108525)
Supplement: Supplementary file 1 [file ijms-24-08525-s001.zip › ijms-2384207-supplementary.pdf]

Supplementary Table S1

Supplementary Table S1. Univariable negative binomial regression models for demographic and clinical factors affecting the antibody (RBD) or T-cell (Spike) immune responses after the second mRNA vaccine dose (T1) or after the booster dose (T2)

			**PwMS vs. HCWs**			
**RBD**		**T1**			**T2**	
	**IRR**	**95%CI**	**p**	**IRR**	**95%CI**	**p**
HCWs	1.00			1.00		
IFN-β	0.77	0.52-1.16	0.209	1.49	0.73-3.05	0.270
Cladribine	0.49	0.30-0.80	**0.004**	0.84	0.31-2.24	0.723
Fingolimod	0.14	0.10-0.21	**<0.001**	0.07	0.04-0.13	**<0.001**
Ocrelizumab	0.02	0.02-0.04	**<0.001**	0.07	0.04-0.14	**<0.001**
Weeks elapsed from the vaccination	0.80	0.74-0.87	**<0.001**	0.79	0.65-0.96	**0.015**
Age	0.99	0.97-1.01	0.163	0.99	0.97-1.02	0.456
Male	0.74	0.48-1.13	0.160	0.96	0.51-1.81	0.904
			**Within PwMS cohort**			
**RBD**		**T1**			**T2**	
	**IRR**	**95%CI**	**p**	**IRR**	**95%CI**	**p**
IFN-β	1.00			1.00		
Cladribine	0.64	0.31-1.32	0.223	0.56	0.13-2.37	0.431
Fingolimod	0.18	0.10-0.34	**<0.001**	0.05	0.02-0.14	**<0.001**
Ocrelizumab	0.03	0.02-0.06	**<0.001**	0.05	0.02-0.15	**<0.001**
Weeks elapsed from the vaccination	1.26	0.74-2.15	0.384	0.91	0.60-1.39	0.676
Age	0.99	0.97-1.02	0.682	0.98	0.95-1.02	0.389
Male	0.69	0.34-1.37	0.283	0.98	0.35-2.76	0.967
BMI	0.98	0.90-1.07	0.683	1.02	0.90-1.16	0.772
Treatment duration	1.09	1.03-1.15	**0.005**	1.09	1.00-1.19	0.053
Disease duration	1.01	0.97-1.04	0.685	1.01	0.96-1.06	0.755
Lymphocytes	1.35	0.73-2.53	0.341	2.39	0.81-7.03	0.115
EDSS	0.75	0.66-0.87	**<0.001**	0.75	0.60-0.94	**0.014**
			**PwMS vs. HCWs**			
**Spike**		**T1**			**T2**	
	**IRR**	**95%CI**	**p**	**IRR**	**95%CI**	**p**
HCWs	1.00			1.00		
IFN-β	0.50	0.31-0.82	**0.006**	0.55	0.27-1.12	0.098
Cladribine	0.26	0.14-0.47	**<0.001**	0.12	0.05-0.30	**<0.001**
Fingolimod	0.01	0.00-0.01	**<0.001**	0.003	0.001-0.01	**<0.001**
Ocrelizumab	0.44	0.27-0.71	**0.001**	0.48	0.24-0.96	**0.037**
Weeks elapsed from the vaccination	0.76	0.70-0.84	**<0.001**	0.84	0.68-1.04	0.103
Age	0.97	0.95-0.99	**0.013**	0.97	0.94-1.01	0.154
Male	1.16	0.71-1.90	0.545	0.65	0.29-1.45	0.292
			**Within PwMS cohort**			
**Spike**		**T1**			**T2**	
	**IRR**	**95%CI**	**p**	**IRR**	**95%CI**	**p**
IFN-β	1.00			1.00		
Cladribine	0.52	0.25-1.10	0.089	0.21	0.07-0.60	**0.004**
Fingolimod	0.01	0.01-0.02	**<0.001**	0.01	0.003-0.01	**<0.001**
Ocrelizumab	0.87	0.46-1.67	0.688	0.87	0.39-1.95	0.741
Weeks elapsed from the vaccination	1.08	0.60-1.97	0.783	1.06	0.77-1.44	0.724
Age	0.98	0.95-1.01	0.294	0.98	0.94-1.02	0.236
Male	0.99	0.47-2.08	0.986	1.18	0.44-3.20	0.743
BMI	1.00	0.94-1.07	0.985	1.02	0.93-1.11	0.696
Treatment duration	1.01	0.95-1.07	0.711	1.02	0.95-1.09	0.644
Disease duration	0.98	0.95-1.02	0.335	0.98	0.93-1.03	0.453
Lymphocytes	2.83	1.33-6.05	**0.007**	11.70	3.80-36.5	**<0.001**
EDSS	0.96	0.83-1.11	0.609	0.85	0.69-1.05	0.135

Footnotes: IRR: incidence rate ratio; PwMS: patients with multiple sclerosis; HCWs: health care workers; CI: confidence interval; RBD: receptor-binding domain; IFN, interferon; BMI: body mass index; EDSS: Expanded Disability Status Scale. In bold are reported the significant values.
